# Evaluation of atorvastatin efficacy and toxicity on spermatozoa, accessory glands and gonadal hormones of healthy men: a pilot prospective clinical trial

**DOI:** 10.1186/1477-7827-12-65

**Published:** 2014-07-12

**Authors:** Hanae Pons-Rejraji, Florence Brugnon, Benoit Sion, Salwan Maqdasy, Gerald Gouby, Bruno Pereira, Geoffroy Marceau, Anne-Sophie Gremeau, Joel Drevet, Genevieve Grizard, Laurent Janny, Igor Tauveron

**Affiliations:** 1 CHU Clermont Ferrand, Laboratoire de BDR: AMP-CECOS, F-63003 Clermont-Ferrand, France; 2 GReD, UMR CNRS 6293 INSERM U1103, Clermont Université, 63000 Clermont-Ferrand, France; 3 Pharmacologie Fondamentale et Clinique de la Douleur, France Inserm, U 1107, Neuro-Dol, Clermont Université, Université d’Auvergne, F-63001 Clermont-Ferrand, France; 4 CHU Clermont-Ferrand, Service d’Endocrinologie-Diabétologie, F-63003 Clermont-Ferrand, France; 5 CHU de Clermont-Ferrand, Délégation à la Recherche Clinique et à l’Innovation (DRCI), F-63003 Clermont-Ferrand, France; 6 CHU Clermont-Ferrand, Biostatistics unit, DRCI, Clermont-Ferrand, France; 7 CHU Clermont-Ferrand, Laboratoire de Biochimie, F-63003 Clermont-Ferrand, France

**Keywords:** Atorvastatin, Human Spermatozoa, Seminal fluid, Cholesterol, Gonadotropins, Testosterone, Normocholesterolemic, Normozoospermic, Healthy men

## Abstract

**Background:**

Recommendations for cardiovascular disease prevention advocate lowering both cholesterol and low-density lipoprotein cholesterol systemic levels, notably by statin intake. However, statins are the subject of questions concerning their impact on male fertility. This study aimed to evaluate, by a prospective pilot assay, the efficacy and the toxicity of a decrease of cholesterol blood levels, induced by atorvastatin on semen quality and sexual hormone levels of healthy, normocholesterolaemic and normozoospermic men.

**Methods:**

Atorvastatin (10 mg daily) was administrated orally during 5 months to 17 men with normal plasma lipid and standard semen parameters. Spermatozoa parameters, accessory gland markers, semen lipid levels and blood levels of gonadal hormones were assayed before statin intake, during the treatment, and 3 months after its withdrawal.

**Results:**

Atorvastatin treatment significantly decreased circulating low-density lipoprotein cholesterol (LDL-C) and total cholesterol concentrations by 42% and 24% (p < 0.0001) respectively, and reached the efficacy objective of the protocol. During atorvastatin therapy and/or 3 months after its withdrawal numerous semen parameters were significantly modified, such as total number of spermatozoa (-31%, p < 0.05), vitality (-9.5%, p < 0.05), total motility (+7.5%, p < 0.05), morphology (head, neck and midpiece abnormalities, p < 0.05), and the kinetics of acrosome reaction (p < 0.05). Seminal concentrations of acid phosphatases (p < 0.01), α-glucosidase (p < 0.05) and L-carnitine (p < 0.05) were also decreased during the therapy, indicating an alteration of prostatic and epididymal functions. Moreover, we measured at least one altered semen parameter in 35% of the subjects during atorvastatin treatment, and in 65% of the subjects after withdrawal, which led us to consider that atorvastatin is unsafe in the context of our study.

**Conclusions:**

Our results show for the first time that atorvastatin significantly affects the sperm parameters and the seminal fluid composition of healthy men.

**Trial registration:**

ClinicalTrials.gov: NCT02094313.

## Background

In the current context of prevention of cardiovascular risks, a decrease of total cholesterol and low-density lipoprotein cholesterol (LDL-C) plasma levels is an important goal for public health [1,2]. Statins are first-choice cholesterol lowering agents [1,3,4]. Meta-analysis of data from randomized controlled trials have demonstrated the higher efficiency of statins on the incidence of cardiovascular morbidity and mortality rates compared with other commonly used lipid-modulating therapies [5]. Atorvastatin, the most commonly used statin, is a synthetic, selective and competitive inhibitor of 3-hydroxy-3-methyl-glutarylcoenzyme A (HMG Co-A) reductase. At the molecular level, statins inhibit cholesterol [6] as well as dolichol and coenzyme Q10 [7,8]. Decreased coenzyme Q10 concentration decreases sperm motility, number of spermatozoa, vitality and increases sperm pathology [8,9].

Sterols are essential in male reproductive physiology, notably for steroidogenesis, spermatogenesis and fertilization [10-12]. During spermatogenesis and epididymal maturation, spermatozoa must undergo a major remodeling of their membrane lipid composition and distribution to acquire progressive motility, final morphology and ability to achieve capacitation and acrosome reaction in the female genital tract [13]. Cholesterol efflux from the plasma membrane is a precursor event of capacitation [14-16], leading to the activation of specific intracellular signal transduction pathways [17-19]. Only capacitated spermatozoa are able to undergo an acrosomal reaction in contact with the zona pellucida before fertilizing the oocyte [20].

Since lipid metabolism disorders are associated with impaired fertility [21] or altered sperm parameters [22], in theory statins could permit improvement in sperm parameters in a context of dyslipidemia. However, concern has always existed that statins might impair testosterone production [23] and potentially affect testicular sperm production and/or quality. In fact, in the scientific and medical literature, the effects of statin therapy on steroid levels and sperm parameters in hypercholesterolaemic men are very heterogeneous, depending on dosage, population and comorbidity context [23-34]. Furthermore, for the past 30 years, studies concerning statins have rarely been exhaustive regarding male fertility, focusing more on steroidogenesis impact than on semen quality. Recently, concerns have been raised about the effect of atorvastatin intake on sperm quality [35]. The most commonly used statin, atorvastatin has never been investigated regarding its influence on male fertility, notably on semen quality.

This study is a prospective pilot assay that evaluates the efficacy and the toxicity of a decrease of cholesterol blood levels induced by atorvastatin on the fertility of healthy men without confounding factors. For the first time the effects of atorvastatin were analyzed on human sperm parameters, accessory gland secretions, semen lipid composition and testosterone and gonadotropins systemic concentrations in normocholesterolaemic and normozoospermic subjects.

## Methods

### Ethics statement

Volunteers were recruited in accordance with the 1975 Helsinki declaration on human experimentation, under a protocol approved for research by a Regional Ethical Committee and the French Agency for the Safety of Health Products (AFSSAPS) and was registered in Clinical Trials Register with the identifier number: NCT 02094313. Written informed consent was obtained from subjects before inclusion in the study.

### Trial design and subjects

The main objective of this pilot study was to estimate the toxicity and the efficacy of atorvastatin on fertility of normocholesterolaemic and normozoospermic men by analyzing its effects on sperm parameters. The secondary objective was to assess the evolution of gonadotropins and total testosterone plasma levels, lipid composition of sperm cells and seminal fluid, spermatozoa capacitation ability and accessory glands markers.

Inclusion criteria were healthy men, 18 to 65 years old, with normal conventional semen parameters and negative semen culture according to the 1999 WHO standards [36] and to David’s criteria for morphology [37], with normal blood lipid profile (total cholesterol < 2.50 g/L, triglycerides < 1.70 g/L, HDL-C > 0.35 g/L and LDL-C < 2.2 g/L) and without known disease or ongoing treatment. Non-inclusion criteria were subjects having a surgical or medical history that might contra-indicate the study, liver disease, prolonged elevation of serum transaminases, lipid parameters not matching with the inclusion criteria, abnormal semen parameters, positive semen culture, cryptorchidism, varicocele, receiving lipid-lowering therapy, or lastly, subjects participating in another clinical study or another experiment over a shorter period than the period of exclusion.

Considering this protocol as a pilot study to evaluate toxicity and efficacy, sample size estimation was fixed according to a one-/multi-stage Fleming design. This design with one group and multi-stages (between 1 and 5) can be seen as filtering steps leading to the “go/no go” decision type. They are among those most used in phase II trials in oncology but remain far more rarely implemented in other areas [38].With type I error α and statistical power (1-β) values of 5% and 90% respectively, 17 subjects were necessary to reject the hypotheses of minimal (p = 0.85) and maximal (p = 0.95) acceptable non-toxicity. If one subject or more presented toxicity, the treatment was considered unsafe.

The safety/toxicity was evaluated by measuring the effects of atorvastatin on sperm parameters according to the 1999 WHO standards [36] (ejaculate volume < 2 ml, sperm count < 20 millions/ml, total motility < 50%, progressive motility < 30%), to David’s criteria for morphology [37] (typical forms < 20%) and to BIOFORMA guidelines [39] for accessory gland markers (fructose ≥ 20 μmol per ejaculate, citric acid ≥ 60 μmol per ejaculate, acid phosphatases ≥ 1234 UI per ejaculate, neutral alpha 1,4 glucosidase ≥ 59 mU per ejaculate, L-carnitine ≥ 390 nmol per ejaculate).

The efficacy was estimated by measuring the lipid lowering action of atorvastatin with expected final levels < 1.5 g/L and < 1 g/L for total cholesterol and LDL-cholesterol respectively, or decreases by 20 and 40% of initials levels of total cholesterol blood and LDL-cholesterol, respectively. To measure the evolution of these parameters 17 subjects were necessary to show the efficacy with a minimal paired difference to be detected of 0.5, with expected standard-deviation of difference of 0.5, correlation coefficient of 0.5, and α value of 5% (two-sided) for a power greater than 90% (1-β = 97%).

Thirty-nine subjects were assessed for eligibility (Flow Diagram is presented in Additional file 1: Figure S1). During the screening visit (visit 0), routine laboratory biochemical tests were carried out, an electrocardiogram was performed, blood pressure, weight and height were measured; physical examination including testis evaluation and semen parameters were analyzed in the Biology of Reproduction Laboratory of the University Hospital of Clermont-Ferrand according to the 1999 WHO standards [36]. Finally, 17 subjects (mean age 24.4 ± 0.9 years) with normal lipid semen parameters were included. The subjects took atorvastatin orally (10 mg/day (d), Tahor©, Pfizer Laboratory) during 5 months allowing the study of atorvastatin effects on human spermatogenesis and epididymal maturation (one cycle requiring approximately 3 months and maximal efficacy of atorvastatin reached within 4 weeks). Blood and semen parameters were measured before (visit 1), during the 5 months of atorvastatin treatment (visit 3), and 3 months after the end of treatment (visit 4), to perform measurements during different cycles of spermatogenesis on a same subject. Biochemical clinical and semen measurements made before treatment were considered as “control baseline measures”. After two months of treatment, a consultation (visit 2) took place to ensure good tolerance of treatment and to control treatment efficiency.

### Chemicals

All chemicals were purchased from Sigma-Aldrich (St Quentin Fallavier, France), unless otherwise indicated.

### Biochemical analyses on blood

Blood was kept on ice in heparin-coated tubes and then centrifuged 15 min at 1500 g at 4°C. Assays were performed on an automated clinical chemistry analyzer (Hitachi Modular; Roche Diagnostics, Meylan, France) based on enzymatic colorimetric (triglycerides, total cholesterol, HDL-C, LDL-C) or electrochemiluminescence assays (total testosterone, FSH, LH) according to the manufacturer’s instructions.

### Semen parameter measurements

The ejaculates were collected by masturbation after 3 to 5 days of abstinence. Immediately after liquefaction semen parameters were evaluated according to the WHO guidelines, 1999 [36]. Sperm morphology was studied according to David’s criteria with the evaluation of Multiple Anomalies Index (MAI) [37]. Sperm motion analysis was realized with a Hamilton-Thorn Sperm Analyzer (HTM Ceros model 12, Hamilton-Thorn Biosciences, Beverly, MA, USA) as previously described [40]. Seminal levels of fructose (seminal vesicle marker), acid phosphatases and citric acid (prostate markers), and α-glucosidase and L-carnitine (epididymal markers) were measured by colorimetric assays as described in the WHO guidelines [36], BIOFORMA guidelines [39] and in a previous work [41].

For semen lipid determination, samples were centrifuged at 500 g for 5 min. The resulting pellet containing the sperm cells was washed twice in Phosphate-buffered Saline (PBS) by centrifugation (500 g, 5 min). The supernatant corresponding to seminal fluid was centrifuged (15 min, 10,000 g, 4°C) to remove cell debris. Lipids present in spermatozoa and in seminal fluid were extracted following a modified Folch procedure [42]. The phospholipids and sterols were separated by high performance thin layer chromatography (HPTLC) using a sequential development system as described in a prior study [43].

**Figure 1:**
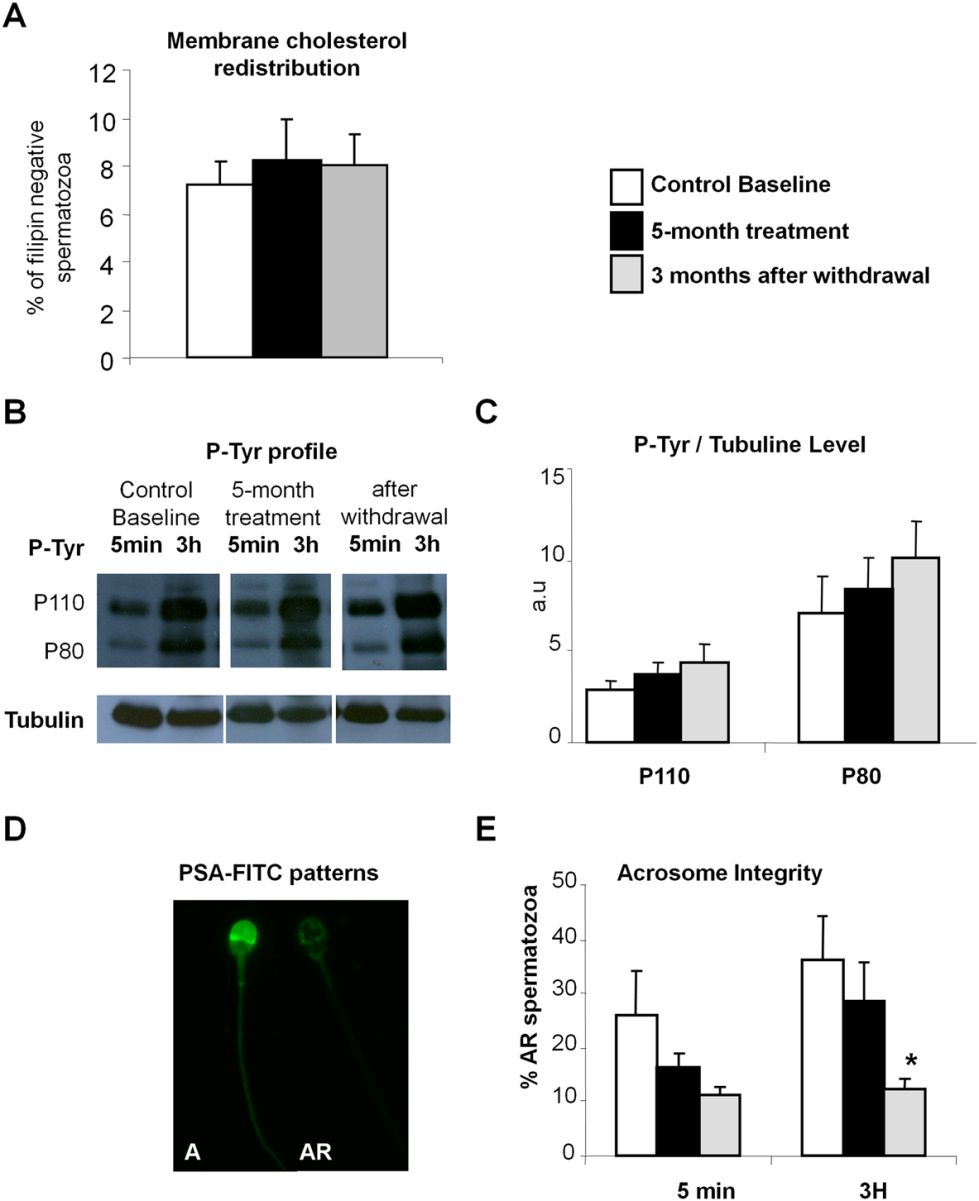
**Atorvastatin effects on membrane cholesterol distribution, P-Tyr and acrosome reaction in spermatozoa.** Membrane cholesterol distribution, P-Tyr and acrosome integrity were analysed in spermatozoa obtained from 17 normocholesterolaemic men before, during and 3 months after the end of treatment as described in Methods. P-Tyr patterns were assessed before (t = 0 h) and after (t = 3 h) incubating spermatozoa in capacitating conditionsd and visualized by western blotting using a monoclonal anti-phosphotyrosine antibody. To calibrate the signal for the amount of sperm protein, the same membranes were reprobed using a monoclonal anti-alpha-tubulin antibody. Acrosome integrity was assessed before (5 min) and after 3 h of incubation in capacitating conditions (3 h), by epifluorescence microscopy (x400) using PSA-FITC as a probe. **A** represents mean ± SEM of the percentage of sperm cells showing a redistribution of cholesterol with filipin labelling absent in the sperm head. Cholesterol distribution was estimated by epifluorescence microscopy (x400) using filipin as a probe. **B** represents typical patterns of protein tyrosine phosphorylation (P-Tyr) and α-tubulin in human spermatozoa. **C** represents P-Tyr signal normalized to the tubulin signal and the ratios were related to the basal signal obtained before incubation. Data are represented as mean ± SEM in arbitrary units (a.u.). **D** represents a fluorescence micrograph showing a sperm cell with an intact acrosome membrane (A pattern: marked fluorescence in the acrosome region) and a sperm cell without an acrosome membrane (AR pattern: no fluorescence or marked fluorescence along the equatorial segment). **E** represents mean ± SEM of the percentage of AR spermatozoa. *indicates values significantly different from those measured before atorvastatin intake with p < 0.05.

Capacitation was estimated by measuring two signaling pathway markers. The first marker, is the early cholesterol redistribution in sperm membranes [44,45]. The second involves the phosphorylation of tyrosine residues (P-Tyr) of terminal protein markers P80 and P110 of cAMP - protein kinase A (PKA) signal pathway. Cholesterol redistribution in sperm membranes was estimated by epifluorescence microscopy (×400) using filipin, as indicated in prior studies [44,45]. Sperm cells with a marked fluorescence in the acrosome region (filipin positive spermatozoa) are not capacitated, while capacitated sperm cells show no fluorescence in the head (filipin negative spermatozoa). For P-Tyr and acrosome integrity assays, spermatozoa were selected using a two-step discontinuous Percoll - HEPES-buffered saline gradient 80-40% (30 min, 1000 g). The 80%-fraction was washed once by centrifugation (500 g, 5 min) in modified Biggers–Whitten–Whittingham medium supplemented with 3 mg/ml BSA and was then incubated in capacitating conditions (37°C, 5% CO_2_). P-Tyr and acrosome integrity were measured after 5 min and 3 h of incubation. P-Tyr of P80 and P110 proteins, was monitored by western-blot using a monoclonal anti-phosphotyrosine antibody (clone 4G10; Upstate Biotechnology Inc, Lake Placid, NY) and a mouse monoclonal anti α-tubulin antibody as indicated in a prior study [46]. The phosphotyrosine signal was normalized to the tubulin signal (Figure 1B and C). The ratios were then related to those obtained before incubation. Acrosome integrity was estimated after spermatozoa fixation, permeabilization in 98% methanol (30 min, - 20°C), and labeling with a freshly prepared solution of *Pisum sativum agglutinin* conjugated to fluorescein isothiocyanate (PSA-FITC, 60 μg/ml final) in PBS. Sperm cells were observed blindly using epifluorescence microscopy (×400). Sperm cells with an intact acrosome membrane show a marked fluorescence in the acrosome region (A pattern, Figure 1D), while those having lost their acrosome membrane are devoid of fluorescence or display a marked fluorescence along the equatorial segment (AR pattern, Figure 1D).

### Statistics

Statistical analyses were performed with Prism 6 (GraphPad Software) using parametric paired tests. The different parameters measured during and 3 months after the end of atorvastatin treatment were compared with initial values (control baseline values), by one-way paired ANOVA with Holm-Sidak multiple comparison post-test when the distribution was normal; if not we applied a nonparametric post-test (Dunn’s Multiple Comparison Test). For each comparison, the corresponding effectiveness was checked to verify the robustness of the analysis.

## Results

Baseline clinical and biochemical characteristics of studied subjects are summarized in Tables 1 and 2. All the subjects were healthy men between 20 and 38 years old (Table 1), with normal renal, hepatic and cardiac functions, body mass index (Table 1) and serum lipid parameters (Table 2) and without known pathology or ongoing treatment. During the 5-month atorvastatin intake period, 15 subjects had a total cholesterol serum level < 1.5 g/L and 16 subjects had a LDL-cholesterol level < 1 g/L, achieving the protocol efficacy goal. We measured a significant decrease of both total cholesterol (-24%, p < 0.0001, Table 2) and LDL-C (- 42%, p < 0.0001, Table 2) blood mean levels. Three months after atorvastatin withdrawal, values of total cholesterol and LDL-C were comparable to those observed before treatment. The serum levels of triglycerides, HDL-C, testosterone, FSH and LH were not affected by atorvastatin (Table 2), nor were the measured blood, hepatic, renal and cardiac markers (data not shown).

**Table 1:** Baseline clinical characteristics of studied subjects

**Baseline clinical characteristics**	
Number of subjects	17
Age (years)	24.35 ± 0.99
BMI	22.74 ± 0.58
Systolic BP (mmHg)	123.53 ± 2.70
Diastolic BP (mmHg)	77.94 ± 0.96
Serum creatinine (μmol/L)	86.76 ± 2.43
Serum glucose (mmol/L)	5.1 ± 0.09
Red blood cells (T/L)	5.1 ± 0.06
Hemoglobin (g/dL)	14.87 ± 0.15
Hematocrite (%)	43.76 ± 0.4
Platelets (G/L)	224.53 ± 9.62
Leukocytes (G/L)	5.75 ± 0.32
Polynuclear neutrophils (G/L)	2.87 ± 0.25
Polynuclear eosinophils (G/L)	0.16 ± 0.03
Polynuclear basophils (G/L)	0.02 ± 0.00
Lymphocytes (G/L)	2.15 ± 0.14
Monocytes (G/L)	0.54 ± 0.04
SGOT (IU/L)	25.65 ± 1.46
SGPT (IU/L)	25.89 ± 3.57
CK (IU/L)	137.88 ± 22.45
GGT (IU/L)	20.82 ± 1.63

Data are presented as the mean ± SEM. BMI: Body Mass Index; BP: blood pressure; SGOT: serum glutamic oxaloacetic transaminase; SGPT: serum glutamic-pyruvic transaminase, CK: Creatine kinase, GGT: Gamma-glutamyl transpeptidase.

**Table 2:** Effects of atorvastatin on blood concentrations of lipids and hormones

	**Control baseline**	**5-month treatment**	**3 months after withdrawal**
**Serum levels of lipids**			
Total cholesterol (g/L)	1.67 ± 0.08	1.27 ± 0.07***	1.71 ± 0.08
Triglycerides (g/L)	0.83 ± 0.06	0.73 ± 0.11	0.93 ± 0.14
HDL-C (g/L)	0.53 ± 0.03	0.52 ± 0.03	0.51 ± 0.03
LDL-C (g/L)	0.98 ± 0.07	0.57 ± 0.05***	0.98 ± 0.07
**Serum levels of hormones**			
FSH (UI/L)	2.85 ± 0.30	2.87 ± 0.33	2.89 ± 0.30
LH (UI/L)	4.47 ± 0.54	5.00 ± 0.37	4.57 ± 0.35
Total testosterone (nmol/L)	22.71 ± 1.56	24.04 ± 1.99	22.81 ± 1.71

Data are presented as mean ± SEM. Seventeen normocholesterolaemic men received 10 mg/d atorvastatin during 5 months as described in Methods. The serum levels of total cholesterol, triglycerides, HDL-C, LDL-C, FSH, LH and total testosterone were measured before, during and 3 months after the end of treatment. ***indicates values significantly different from those obtained before treatment with p < 0.001.

The reduction of cholesterol level in serum did not significantly affect semen cholesterol and phospholipid levels, whether in spermatozoon or in seminal fluid (Table 3). However, we noted that the cholesterol/phospholipids ratio tended to decrease both in sperm cells and in seminal fluid during the treatment.

**Table 3:** Effects of atorvastatin on human semen parameters and lipids composition

	**Control baseline**	**5-month treatment**	**3 months after withdrawal**
**Conventional semen parameters**			
Semen volume (ml)	3.9 ± 0.4	4.0 ± 0.6	3.5 ± 0.4
Semen pH	8.0 ± 0.1	7.9 ± 0.1	8.1 ± 0.2
Sperm concentration (10^6^ spz/ml)	146.1 ± 17.8	119.1 ± 15.6	109.9 ± 14.8
Sperm number (10^6^ spz/ejaculate)	540.5 ± 83.3	449.1 ± 75.6	375.0 ± 60.5*
Vitality (%)	85.2 ± 1.5	78.8 ± 1.7**	77.1 ± 2.7*
Total motility (%)	60.9 ± 1.8	65.5 ± 2.2*	62.7 ± 2.5
Progressive motility (%)	57.9 ± 1.8	58.6 ± 2.2	58.0 ± 2.8
VSL (μm/s)	50.4 ± 2.9	47.5 ± 1.5	50.6 ± 2.5
VCL (μm/s)	84.6 ± 2.5	80.8 ± 2.3	87.1 ± 3.2
Linearity (%)	61.1 ± 2.2	58.8 ± 1.4	57.9 ± 2,0
ALH (μm)	3.7 ± 0.2	3.6 ± 0.1	3.8 ± 0.2
Sperm abnormalities (%)	64.5 ± 2.6	63.5 ± 2.4	66.4 ± 2.4
Head abnormalities	66.1 ± 4,0	64.8 ± 3.5	73.5 ± 4.6^‡^
Excess residual cytoplasm	3.1 ± 0.6	5.8 ± 1.3	5.2 ± 0.9
Neck and midpiece abnormalities	13.3 ± 1.9	13.0 ± 2.2	17.7 ± 1.7*
Tail abnormalities	6.6 ± 1.0	4.7 ± 0.8	6.9 ± 1.2
MAI	1.6 ± 0.18	1.41 ± 0.03	1.54 ± 0.05^‡‡^
**Cholesterol and phospholipid concentrations in Spermatozoa (nmol/10**^**8**^**)**			
Cholesterol	80.94 ± 6.08	95.47 ± 9.88	93.32 ± 8.09
PE	50.46 ± 6.10	61.82 ± 6.32	57.37 ± 6.91
PC	120.55 ± 17.84	158.00 ± 18.09	142.97 ± 16.83
SM	34.48 ± 4.11	42.56 ± 6.47	32.61 ± 2.42
Cholesterol/(PE + PC + SM) ratio	0.46 ± 0.04	0.39 ± 0.03	0.44 ± 0.05
**Cholesterol and phospholipid concentrations in Seminal fluid (μmol/ml)**			
Cholesterol	34.38 ± 2.55	35.24 ± 3.00	34.48 ± 3.33
PE	5.52 ± 0.64	6.10 ± 0.73	7.95 ± 1.75
PC	6.95 ± 0.78	7.21 ± 0.95	7.90 ± 2.09
SM	12.58 ± 1.38	12.71 ± 1.42	12.07 ± 1.14
Cholesterol/(PE + PC + SM) ratio	1.44 ± 0.06	1.40 ± 0.06	1.36 ± 0.07

Data are presented as the mean ± SEM. Seventeen normocholesterolaemic men received 10 mg/d atorvastatin during 5 months as described in Methods. The semen parameters and cholesterol and phospholipid concentrations were measured before (control baseline values), during and 3 months after the end of treatment.

VSL: straight line velocity; VCL: curvilinear velocity; ALH: amplitude of lateral head displacement; MAI: Multiple Anomalies Index; PE: phosphatidylethanolamine; PC: phosphatidylcholine; SM: sphingomyelin. *and **indicate that the value is significantly different from those obtained before treatment with p < 0.05 and p < 0.01 respectively. ^‡^ and ^‡‡^ indicate that the value is significantly different from the value measured during atorvastatin uptake with p < 0.05 and p < 0.01 respectively.

**Figure 2:**
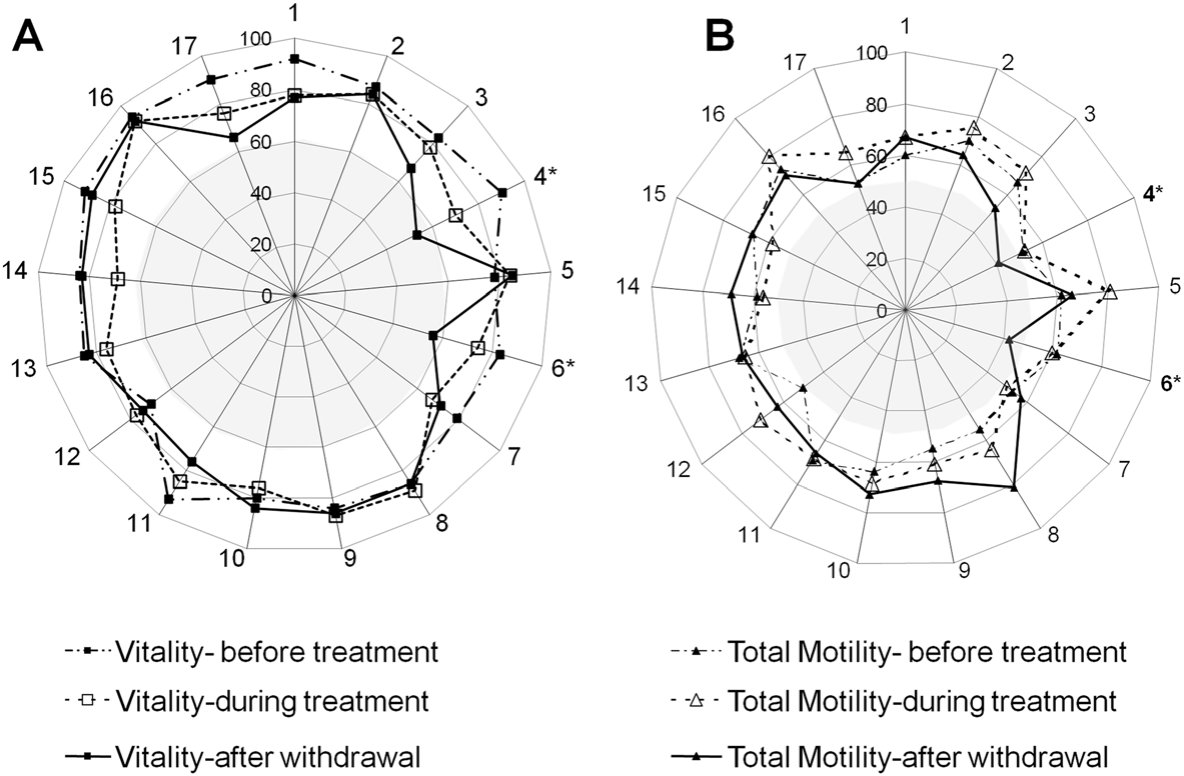
**Effects of atorvastatin on human sperm vitality and progressive motility.** Percentages of viable **(A)** and total motile **(B)** spermatozoa of the seventeen subjects are represented in the spider graphs before (control baseline values), during and 3 months after the end of treatment. *indicates patients having altered sperm parameters according to WHO standards.

Mean values of semen parameters were significantly altered by atorvastatin intake (Table 3). Total sperm number (-31%) and vitality (-9.5%) were significantly reduced, notably after treatment withdrawal (p < 0.05). The teratozoospermia Multiple Anomalies Index (MAI) and the number of head, neck and midpiece abnormalities were significantly increased (p < 0.05), even if the proportion of morphologically abnormal spermatozoa was not globally modified. Conversely, total motility was slightly but significantly improved during treatment (65.5 ± 2.2% during *vs.* 60.9 ± 1.8% before, p < 0.05), with no effect either on progressive motility or on sperm motion parameters. When the effects were analyzed per subject, 2 out of 17 men were necro- and asthenozoospermic according to the WHO 1999 standards, notably after withdrawal of treatment (Figure 2A and B), exceeding the safety limits of the study.

**Figure 3:**
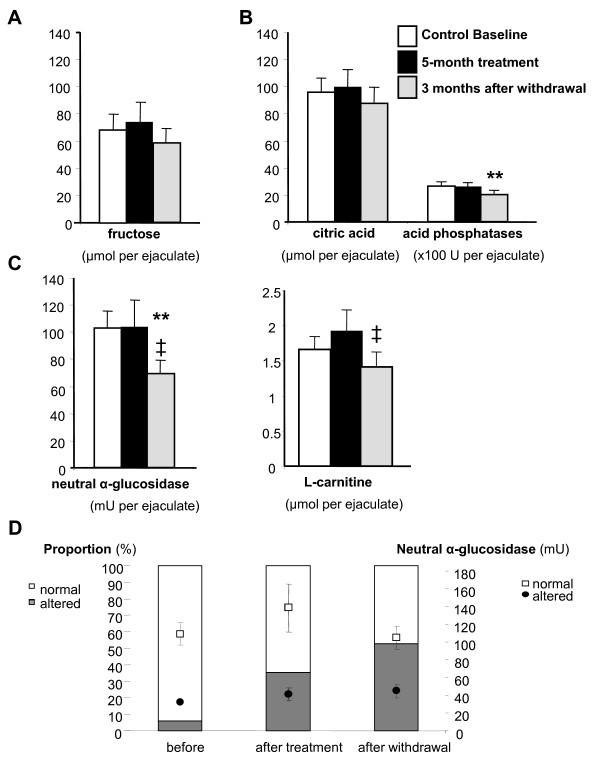
**Atorvastatin effects on markers of the accessory sex glands.** The seminal levels of fructose **(A)** (seminal vesicle marker), acid phosphatases and citric acid (**B** (prostate markers), and alpha-glucosidase and L-carnitine **(C and D)** (epididymal markers) were measured in 17 normocholesterolaemic men before, during and 3 months after the end of treatment, as described in Methods. In A, B and C, bar represents mean ± SEM. **indicates values significantly different from those obtained before atorvastatin intake with p < 0.01 and ‡ indicates values significantly different from those measured during atorvastatin treatment with p < 0.05. In D, bars (white and coloured) and symbols (points and squares) represent mean ± SEM and percentages respectively of the subjects having normal (white) and altered (coloured) values of neutral α-glucosidase activity before, during and 3 months after the end of treatment.

Markers of the accessory sex glands were also perturbed. The amount of prostatic acid phosphatases was significantly reduced 3 months after atorvastatin withdrawal (p < 0.01 *vs.* control value, Figure 3B). Similarly, the levels of epididymal neutral α-glucosidase and L-carnitine were significantly decreased (p < 0.01 and p < 0.05 for α-glucosidase and L-carnitine, respectively, Figure 3C). Regarding individual impact, one patient presented an abnormal value for neutral α-glucosidase (6% value, < 59 mU, Figure 3D) before the beginning of treatment according to BIOFORMA standards. During the treatment 35% (6 subjects) showed altered activity of neutral α-glucosidase with a mean value of 40.0 ± 7.6 mU. Three months after the end of therapy, more than half of the subjects presented an abnormal value of neutral α-glucosidase (53%, 9 subjects Figure 3D) with an activity mean of 43.8 ± 7.8 mU. Atorvastatin effects on neutral α-glucosidase again exceeded the safety limits fixed by the study.

To further evaluate the fertilizing ability of spermatozoa, capacitation and acrosome reaction were measured in capacitating conditions. Spermatozoa capacitation was evaluated by measuring early markers such as cholesterol depletion from plasma membrane and terminal markers of signaling pathways corresponding to the P-Tyr levels of proteins P80 and P110. Atorvastatin treatment had no effect on the proportion of filipin negative spermatozoa (Figure 1A) nor on the P-Tyr level of the two protein markers of capacitation P110 and P80 (Figure 1B and C). However, it was observed that the level of P-Tyr tended to increase during treatment and 3 months after atorvastatin withdrawal. The proportion of spontaneous acrosome-reacted (AR, Figure 1D) spermatozoa tended to be reduced by the cholesterol-lowering therapy (16.1 ± 3.0% during *vs.* 26.1 ± 7.8% before treatment, 5 min, Figure 1E) and after 3 h of incubation under capacitating conditions (28.3 ± 7.4% *vs*. 36.1 ± 8.5% before atorvastatin intake, Figure 1E). This trend was also observed 3 months after the end of the therapy and became significant for the AR proportion obtained after 3 h of incubation under capacitating conditions (12.3 ± 2.0%, p < 0.05 in comparison with values before atorvastatin treatment).

## Discussion

This is the first study evaluating atorvastatin effects on semen quality and testicular steroidogenesis regulation in normocholesterolaemic, young and healthy men. This work is a prospective non-controlled pilot assay evaluating atorvastatin efficacy and toxicity on human semen parameters. A 5-month atorvastatin intake (10 mg/d) induced a significant decrease in serum levels of total cholesterol and LDL-C achieving the efficacy goal of the protocol. Atorvastatin treatment affected semen parameters significantly. It also decreased the total number of spermatozoa in ejaculate and sperm vitality, increased morphological abnormalities and total motility, and altered acrosome reaction kinetics. Seminal concentrations of acid phosphatases, α-glucosidase and L-carnitine were also decreased. All these results hint at deleterious effects of atorvastatin on testicular, prostatic and epididymal functions.

At the end of the study, 9 subjects (53%) had an alteration of at least one semen marker; two men were necro-asthenozoospermic and had a low value of neutral α-glucosidase. We had specified that if one subject or more presented toxicity on sperm or accessory gland markers, the treatment was considered unsafe. The results obtained led to the decision to stop the protocol and to conclude in the efficacy but also the toxicity of atorvastatin for semen parameters. To further evaluate the fertilizing ability of spermatozoa, capacitation and acrosome reaction were measured in capacitating conditions. To our knowledge, fertilizing ability has never been studied in the context of a cholesterol-lowering therapy in humans. Atorvastatin had no significant effect on early (cholesterol depletion from plasma membrane) or terminal markers (P-Tyr levels of proteins P80 and P110) [19,47] of capacitation. Nevertheless, the proportion of acrosome-reacted spermatozoa was significantly decreased by atorvastatin intake.

Our study showed that atorvastatin therapy does not alter cholesterol and phospholipid composition of human spermatozoa and seminal fluid. This is consistent with our previous study showing that normo- and hypercholesterolaemic men had similar amounts of cholesterol and phospholipids in semen [41]. The same observations were made in the rabbit [48-50], with, however, a modification of sterol distribution in acrosome and plasma membranes, and significant alterations of sperm parameters. In our study, we did not observe any significant modification of cholesterol distribution in the human sperm head as shown by filipin fluorescence, even if acrosome reaction kinetic was slightly altered. These discrepancies could be explained by differences in cholesterol regulation between the different models. Nevertheless, we can exclude yhe postalte that atorvastatin could affect other sterols in the human sperm membrane.

Similarly, modifications of systemic cholesterol metabolism do not significantly modify cholesterol and phospholipid levels in semen. These data could explain the absence of effects of atorvastatin on systemic levels of total testosterone and gonadotropins. Nevertheless, sperm production and quality were decreased and prostatic and epididymal markers were altered. Such data indicate clearly that atorvastatin therapy affects testicular, epididymal and prostatic functions. Testis, epididymis [51,52] and prostate [53] are androgen-dependent organs. Epididymal secretion of α-glucosidase, specifically, is androgen - dependent [54,55]. Since gonadotropin and testosterone plasma levels were not affected, we can exclude a direct effect of peripheral blood sexual hormones. Furthermore, the main income of testosterone in the epididymis is through the efferent ducts [51,52]. An hypothesis could be that atorvastatin induced a local and minor alteration of testosterone and/or dihydrotestosterone (DHT) homeostasis in the genital tract that does not impact testosterone and gonadotropin systemic levels. Epididymal, testicular or spermatic cord blood measures of androgen levels are difficult to perform in the humans to verify this hypothesis. Maybe the seminal fluid assays of testosterone and DHT would be indicative. Use of animal models could also allow deepening the effect of atorvastatin on male genital tract and notably on testicular steroïdogenesis. This should allow at least a measurement of the intratesticular level of androgens.

We could also consider that atorvastatin might induce local inflammation or oxidative stress with persistent effects on prostatic and epididymal secretion. A recent study has characterized prostatic acid phosphatases regulation in androgen-dependent and -independent manner [56], suggesting that it could be considered that statin effects on the genital tract could be more complex than an androgen-mediated action. Moreover, most of the effects of atorvastatin were observed 3 months after stopping the treatment, despite the return of cholesterol and LDL-C to normal levels. It would have been interesting to follow the subjects for a longer time after atorvastatin withdrawal to determine when the parameters returned to baseline.

This persistent effect could indicate that atorvastatin effects on the genital tract could be also mediated by a messenger or an intermediary that persists after the end of therapy. In 2005, Niederberger raised the question of the adverse effect of atorvastatin on human fertility [35]. One hypothesis to explain this action is that atorvastatin induces a decrease in ubiquinone oxido-reductase (Coenzyme Q10) level. The enzyme represents an important intracellular antioxidant for spermatozoa in the seminal fluid and its concentration in seminal fluid is positively correlated with sperm motility [57,58]. Recently, it was proposed that statins could act by three major intracellular mechanisms implicating insulin signaling transduction pathway, ATP and calcium regulation [8]. Atorvastatin could also affect important downstream products such as Coenzyme Q10, farnesyl pyrophosphate, geranylgeranyl pyrophosphate, and dolichol. A last hypothesis could be that atorvastatin acts *via* these different products leading to alterations of intracellular signaling pathways in genital tract tissue or sperm cells, associated or not with an oxidative stress. Once again, animal models could be an interesting perspective to characterize atorvastatin effects on the genital tract.

A major deleterious effect of atorvastatin was observed on secretory gland markers, notably on epididymal markers. Purvis *et al.,* in 1992 ([23,59], Additional file 2: Table S1) measured no effect of simvastatin intake (3.5 months at 40 mg/d) on human accessory gland markers. However, it was established that statins can affect the prostate, notably prostate-specific antigen (PSA) serum levels [60,61], and can act within prostate cell membrane on discrete regions known as lipid rafts [6,62]. Furthermore it was demonstrated that statins, notably simvastatin, have an effect on the synthesis and secretion of cholesterol by human PC3prostate cancer cells *via* prostasomes [63,64]. In contrast to acid phosphatases, the seminal levels of citric acid (another prostate marker) and sterols (whose main source is the prostate) remained unchanged. These data suggest that atorvastatin could affect specifically the synthesis and/or the secretion of acid phosphatases, possibly by targeting specific prostatic cells or by a specific signaling pathway. A previous study has demonstrated that the seminal L-carnitine level was significantly increased in men with hypercholesterolemia [41]. The epididymis is the site of sperm maturation and acquisition of linear and progressive motility. We found moderate but significant changes in the morphology and motility of sperm, in accordance with an effect of atorvastatin on the primary functions of the epididymis.

The main objective of this study was to determine specific effects of atorvastatin intake on semen parameters and hormonal regulation of young men in the context of secondary cardiovascular prevention. Previous studies (for details see Additional file 2: Table S1) have shown deleterious effects of simvastatin or pravastatin on human sperm parameters [65-67]. A few others measured no effect of pravastatin or simvastatin intake on sperm quality [28,59]. In all these studies, statin effects were of low amplitude and values measured after therapy remained within normal values, indicating no clinical deleterious impact on human sperm quality.

Similarly, some authors reported an association between statin therapy and hypogonadism [25,27,33,34,60,68], while others did not, notably at low or moderate doses (between 10 and 40 mg/d) of simvastatin [29,59,65], pravastatin [28,29,65,66,69,70], lovastatin [67] or atorvastatin [30,71]. Since these different statins have different hydrophilic properties, it cannot be excluded that their pharmacokinetic or physicochemical properties have an impact on their effects. Recently, a meta-analysis of placebo randomized controlled trials of statins demonstrated that statin therapy decreased testosterone by 0.66 in middle-aged men with hypercholesterolemia [23]. Nevertheless, this average change is limited, while the range of normal values for testosterone is quite wide. Moreover, male hypogonadism and dyslipidaemia have been associated with different concomitant morbid factors including age, renal disease, type 2 diabetes, obesity, liver cirrhosis, metabolic syndrome or erectile dysfunction [23,30,31,33,68,72-76]. These co-morbidity factors could be confounding elements and, at least partially, responsible for the deleterious effects on androgen regulation and sperm quality. In this study, we measured the effects of statins on the fertility of young, healthy and normocholesterolaemic subjects without confounding factors. Only one previous study analyzed the effects of statins on healthy men, and only steroidogenesis was explored [77].

Our work demonstrates that atorvastatin therapy, at 10 mg daily, had no effect on total testosterone and gonadotropin serum level but affected significantly sperm parameters of young and healthy men and was considered as deleterious in the context of our protocol. Yet, in view of our results in this young population, it may be considered that the effects could be more pronounced among older men specifically if less healthy. For this pilot assay, control baseline values were measured for each subject before the beginning of the atorvastatin treatment, rather than include a placebo-treated group allowing us to limit the high inter-individual variability and to include a relatively small numbers of subjects. A perspective will be to perform a randomized placebo-controlled assay with a larger cohort and a longer study time to confirm and deepen atorvastatin effects on men fertility.

## Conclusions

In conclusion, our study shows for the first time that the intake of atorvastatin by healthy and normocholesterolaemic subjects significantly affected their sperm parameters (vitality, number, motility, morphology and acrosome reaction) and changed their seminal fluid composition. These consequences occurred after only 5 months of treatment. Considering the long duration of statin treatment, for which the clinical benefit with respect to cardiovascular diseases is beyond question, potential negative consequences on reproductive function should be taken into account when deciding to initiate such a treatment, notably for young adults.

## Competing interests

The authors declare that they have no competing interests.

## Authors’ contributions

HPR participated in the design of the study and its coordination, carried out semen assays and drafted the manuscript. FB participated in the design of the study, its coordination, in the inclusion of the patients and helped to draft the manuscript. BS participated in the design of the study, carried out the semen lipid and filipin assays and helped to draft the manuscript. SM carried out the carnitine assay. GG participated in the design of the study and helped to draft the manuscript. BP performed the statistical analysis and helped to draft the manuscript. GM performed blood biochemical analyses. ASG performed the physical examination of the patients, including testis evaluation. JRD helped to draft the manuscript. GG conceived the study and participated in its design and coordination and helped to draft the manuscript. LJ conceived the study and participated in its design and in the inclusion of the patients, and helped to draft the manuscript. IT conceived the study and participated in its design and coordination and helped to draft the manuscript. All authors read and approved the final manuscript.

## Supplementary Material

Additional file 1: Figure S1 Flow diagram. After a screening visit (visit 0) allowing inclusion and exclusion of subjects by the measurement of the clinical, blood and semen baseline parameters, serum and semen parameters of subjects were assayed in the laboratory during 4 visits corresponding to the beginning of atorvastatin treatment (10mg daily) (visit 1); a control visit to ensure good tolerance to treatment and its efficiency after 2 months of therapy (visit 2) and the measurement of the effects of a 5-month atorvastatin intake (visit 3) and the residual effects after 3 months of treatment withdrawal (visit 4). Click here for file

Additional file 2: Table S1 Prospective studies analyzing statin effects on human gonadal steroidogenesis and semen quality. *after 6 months of therapy, ref.: reference, treat.: treatment, normochol.: normocholesterolæmic; hyperchol.: hypercholesteroleamic. Test: testosterone, Num: sperm number, Motil: sperm motility; Morph: sperm morphology; Acc. glands: Accessory glands; Cap: capacitation; AR: Acrosome reaction. Click here for file
