# Supplementary material for: Evaluation of atorvastatin efficacy and toxicity on spermatozoa, accessory glands and gonadal hormones of healthy men: a pilot prospective clinical trial
Source: Reprod Biol Endocrinol. 2014 Jul 12;12:65. doi: 10.1186/1477-7827-12-65 (PMC4114109; doi:10.1186/1477-7827-12-65)
Supplement: Additional file 1: Figure S1 — Flow diagram. After a screening visit (visit 0) allowing inclusion and exclusion of subjects by the measurement of the clinical, blood and semen baseline parameters, serum and semen parameters of subjects were assayed in the laboratory during 4 visits corresponding to the beginning of atorvastatin treatment (10mg daily) (visit 1); a control visit to ensure good tolerance to treatment and its efficiency after 2 months of therapy (visit 2) and the measurement of the effects of a 5-month atorvastatin intake (visit 3) and the residual effects after 3 months of treatment withdrawal (visit 4). [file 1477-7827-12-65-S1.pdf]

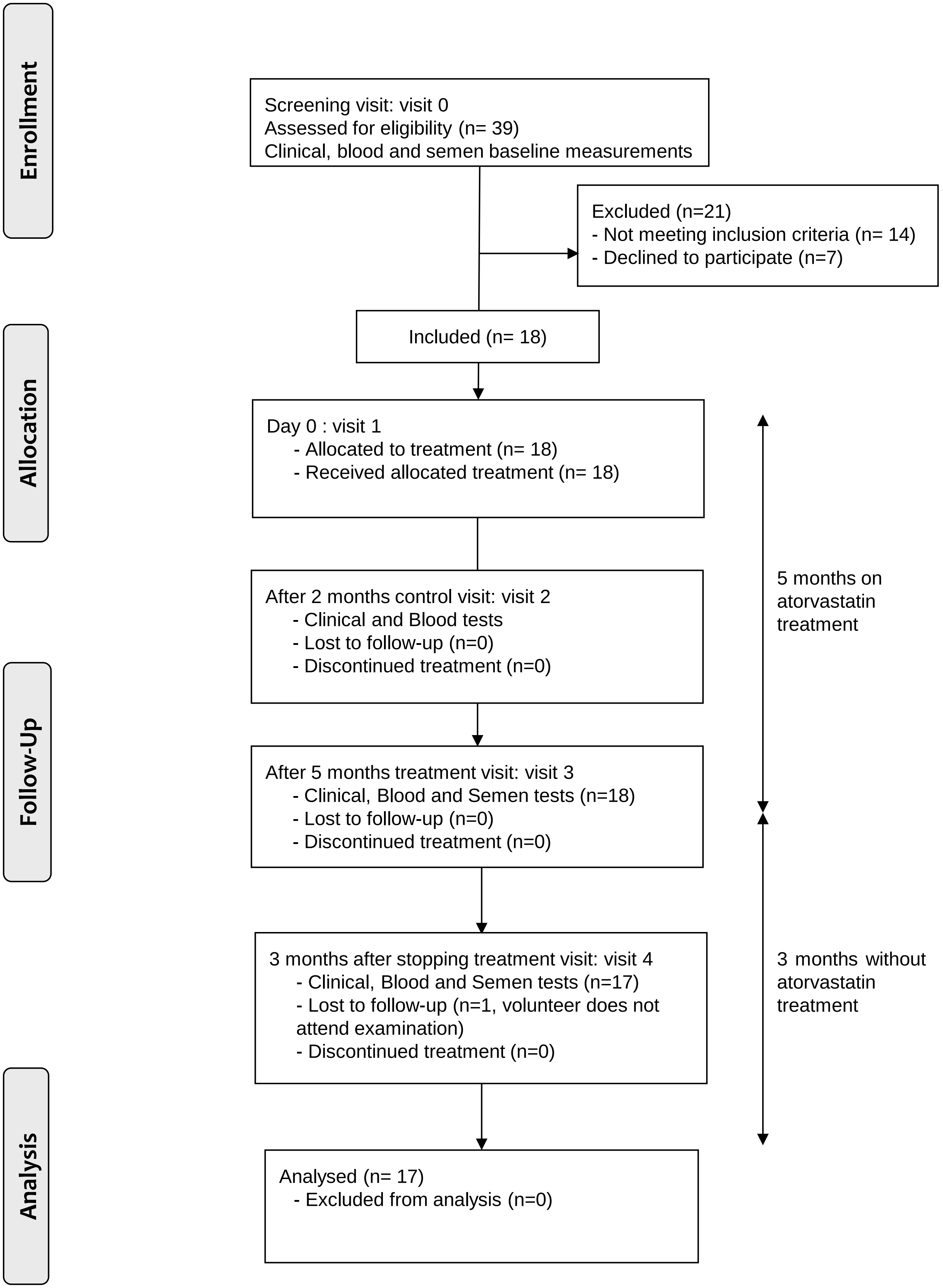
Enrollment
Screening visit: visit 0
Assessed for eligibility (n= 39)
Clinical, blood and semen baseline measurements
Excluded (n=21)
- Not meeting inclusion criteria (n= 14)
- Declined to participate (n=7)
Included (n= 18)
Allocation
Day 0 : visit 1
- Allocated to treatment (n= 18)
- Received allocated treatment (n= 18)
5 months on atorvastatin treatment
Follow-Up
After 2 months control visit: visit 2
- Clinical and Blood tests
- Lost to follow-up (n=0)
- Discontinued treatment (n=0)
After 5 months treatment visit: visit 3
- Clinical, Blood and Semen tests (n=18)
- Lost to follow-up (n=0)
- Discontinued treatment (n=0)
3 months after stopping treatment visit: visit 4
- Clinical, Blood and Semen tests (n=17)
- Lost to follow-up (n=1, volunteer does not attend examination)
- Discontinued treatment (n=0)
3 months without atorvastatin treatment
Analysis
Analysed (n= 17)
- Excluded from analysis (n=0)
