# Supplementary material for: Evaluation of atorvastatin efficacy and toxicity on spermatozoa, accessory glands and gonadal hormones of healthy men: a pilot prospective clinical trial
Source: Reprod Biol Endocrinol. 2014 Jul 12;12:65. doi: 10.1186/1477-7827-12-65 (PMC4114109; doi:10.1186/1477-7827-12-65)
Supplement: Additional file 2: Table S1 — Prospective studies analyzing statin effects on human gonadal steroidogenesis and semen quality. *after 6 months of therapy, ref.: reference, treat.: treatment, normochol.: normocholesterolæmic; hyperchol.: hypercholesteroleamic. Test: testosterone, Num: sperm number, Motil: sperm motility; Morph: sperm morphology; Acc. glands: Accessory glands; Cap: capacitation; AR: Acrosome reaction. [file 1477-7827-12-65-S2.pdf]

ref.	n	age range years	subject lipids statut	treat. period months	statins molecule	statin dose mg/d	Effets on human Hormones Test	LH	FSH	Semen Num	Motil	Morp	Acc. glands	Cap /AR	Semen lipids
**Present study**	**17**	**20-38**	**normochol. men**	**5/ 3 after**	**atorvastatin**	**10**	**=**	**=**	**=**	↓	↑	↓	↓	=/↓	=
Santini et al., 2003	24	54-72	type 2 diabetic hyperchol. men and women	3	atorvastatin	20	=	=	=						
Bôhm et al., 2004	22	56-76	hyperchol. men and women	3	pravastatin/ placebo	40	=	=	=						
Hyypa et al., 2003	120	35-64	hyperchol. men	3	simvastatin/ placebo	20	↓								
Dobs et al., 2000a	164	21-55	hyperchol. men	3	simvastatin/ placebo	80	↓	=	=						
Dobs et al., 2000b	159	21-55	hyperchol. men	6	pravastatin/placebo<br>simvastatin/placebo<br>simvastatin/placebo	40<br>20<br>40	=<br>=<br>=	=<br>=<br>=	=<br>=<br>=	=<br>=<br>=	=<br>=<br>=	=<br>↑<br>=			
Stein et al., 1998	521	45-66	hyperchol. men and women	6	simvastatin	40 to 80	↓	=	=						
Bernini et al., 1998	10	31-60	hyperchol. men	6	pravastatin	20	=	=	=	=	=	=			
Azzarito et al., 1996	8	54-58	type IIA hyperchol. men	3-12	simvastatin	20	↓*	=	=						
Travia et al. 1995	24		hyperchol. men	6-36/ 2 after	simvastatin<br>pravastatin	40<br>40	=<br>=								
Rossato et al., 1993	18	42-58	hyperchol. men	1-12	simvastatin	10	=	=	=						
Dobs et al., 1993	64	21-70	hyperchol. men and women	6-2/ 12 after	pravastatin/placebo + cholestyramine	20<br>80	=<br>=	=<br>=	=<br>=	↓ *<br>=	↓ *<br>↓	=<br>=			
Purvis et al., 1992	19	20-49	hyperchol. men	3,5	simvastatin/placebo	40	=	=	=	=	=	=	=		
Azzarito et al., 1992	10	51-66	hyperchol. men and women	12	simvastatin	20 to 40	=								
Jay et al., 1991	23		hyperchol. men and women	3/ 6 after	pravastatin/cholesty ramine/placebo		=	=	=						
Farnsworth et al., 1987	16	28-66	hyperchol. men	4	lovastatin/neomycin /placebo	40	=	=	=	=	↓				
Tobert et al. 1982	59	18-45	normochol. men	1	lovastatin/placebo	6.25 to 50	=								
